# Resting-state hippocampal asymmetry as a marker for memory and olfactory deficit in parkinson’s disease

**DOI:** 10.1038/s41598-025-29976-2

**Published:** 2025-11-26

**Authors:** Tom Eek, Thomas A.W. Bolton, Nil Dizdar, Maria Larsson, Fredrik Lundin, Charalampos Georgiopoulos

**Affiliations:** 1 Department of Biomedical and Clinical Sciences, Linköping University, Linköping, Sweden; 2 Center for Medical Image and Visualization, Linköping University, Linköping, Sweden; 3 Connectomics Laboratory, Department of Radiology, Centre Hospitalier Universitaire Vaudois (CHUV), Lausanne, Switzerland; 4 Department of Psychology, Gösta Ekman Laboratories, Stockholm University, Stockholm, Sweden; 5 Diagnostic Radiology, Department of Clinical Sciences, Medical Faculty, Lund University, Lund, Sweden; 6 Department of Neurology, Linköping University Hospital, Linköping, 581 85 Sweden

**Keywords:** Parkinson’s disease, Co-activation pattern analysis, Asymmetric hippocampal activity, Memory, Odor identification, Diseases, Neurology, Neuroscience

## Abstract

Memory decline is a central cognitive symptom in Parkinson’s Disease (PD). While task-fMRI studies link hippocampal activity (AHA) to poorer memory and olfactory performance, this relationship during rest remains understudied. The objectives of this study are to examine differences in resting-state hippocampal networks, explore the occurrence of reduced AHA within these networks, and investigate its impact on memory and olfaction in PD. Thirty-nine PD patients awaiting evaluation for device-aided Parkinson therapy and 46 healthy controls (HC) underwent resting-state fMRI (rs-fMRI). PD patients also completed a memory and olfactory assessment. Co-activation pattern (CAP) analysis was performed on the rs-fMRI data. Our results demonstrated reduced activity in two hippocampal networks in PD: Network 1, incorporating the visual cortex, cerebellum, superior parietal lobule, and precuneus, and Network 5, incorporating parts of the central executive network. PD subgroups with reduced AHA in Network 1 and 5 performed significantly worse on tests of auditory-verbal short-term, long-term and recognition memory, as well as odor identification. In conclusion, within specific resting-state hippocampal networks, reduced AHA in PD is linked to poorer auditory-verbal memory and odor identification.

## Introduction

In addition to the well-documented motor symptoms of Parkinson’s Disease (PD), PD also involves a range of severely debilitating non-motor symptoms^1^. Among these, impaired sense of smell (hyposmia) and memory are particularly common. Approximately 90% of PD patients suffer from hyposmia, often detectable before motor symptoms onset, and 50% develop mild cognitive impairment within five years of diagnosis^2,3^. Hyposmia significantly impacts daily activities, such as cooking and enjoying food. Additionally, depression is more prevalent and severe in hyposmic patients compared to their normosmic counterparts^4,5^. Memory impairment has been associated with lower quality of life, and cognitive symptoms play a key role in the decision to place PD patients in nursing homes^6,7^. Moreover, olfaction and memory are strongly connected, with the deterioration of one function indicating a potential decline in the other^8^. This connection is also reflected in the engagement of the hippocampus in both functions. For instance, several studies have shown a relationship between hippocampal activity and successful odor recognition memory (ORM), between progression of hippocampal atrophy in PD and episodic memory decline, as well as between reduced hippocampal connectivity and the ability to identify odors^9–12^.

In the advanced phase of the disease, characterized by worsening motor and non-motor symptoms mainly due to the progressive degeneration of dopaminergic neurons, PD patients experience the “on-off phenomenon”. This refers to rapid and unpredictable motor fluctuations even after repeated optimization of oral dopaminergic medications^13^. Consequently, device-aided Parkinson therapies (DAPT) are required to achieve continuous dopaminergic stimulation and, as a result, symptom relief. Although DAPT, such as deep brain stimulation of the subthalamic nucleus (STN-DBS), is highly effective, particularly in treating motor symptoms, approximately 20% of PD patients show further cognitive decline following STN-DBS^14^. Given that PD patients are already cognitively vulnerable and that up to 80% will eventually develop Parkinson’s Disease Dementia, it is crucial to explore new methods for characterizing patients who may be at increased risk for cognitive decline, in order to inform the selection of appropriate DAPT^1^.

The hemispheric asymmetry reduction in older adults (HAROLD) model is supported by numerous studies, demonstrating that older adults, compared to younger individuals, exhibit less lateralized brain activity, particularly in the prefrontal cortex^15^. This reduction in asymmetry has been observed during tasks involving episodic, semantic, short-term, and working memory. It is thought to be associated with compensatory processes for age-related cognitive decline by recruiting additional brain regions (compensation theory) and/or to a diminished capacity to recruit specialized brain areas (dedifferentiation theory)^15^. A recent task-fMRI study from our group showed a similar pattern in PD patients compared to healthy controls (HC). Specifically, PD patients exhibited significantly reduced asymmetric hippocampal activity within certain hippocampal networks during an ORM-task. Furthermore, PD patients with reduced asymmetric hippocampal activity (AHA) demonstrated impaired ability to identify distractor odors, indicating a potential link between AHA and ORM performance^16^.

Nevertheless, several aspects of reduced AHA in PD have remained understudied: whether it also occurs during rest, and whether resting-state AHA is linked to memory and olfactory abilities beyond ORM. Resting-state fMRI (rs-fMRI) offers several advantages compared to task-fMRI. In rs-fMRI, the scanning procedure is simpler, minimizing the possibility of gathering incorrect data^17^. For instance, responses during task-fMRI are usually delivered by button-pushing which can be challenging for PD patients and therefore a source of errors. It is also more convenient, as less patient-proximal equipment is required. Additionally, rs-fMRI adopts a more data-driven and less theory-based approach, focusing on spontaneous brain activity and connectivity^18^. This is suitable for relatively unexplored area, such as the relationship between changes in AHA, memory, and olfaction in PD, which may initially benefit from exploratory research.

Given this background, our study focuses on mapping hippocampal connectivity patterns related to memory and olfactory performance, potentially serving as a biomarker for cognitive impairment in PD patients experiencing the “on-off phenomenon”. The early detection of cognitive impairment in these patients is crucial since they are awaiting evaluation for DAPT. The study aims are as follows: (1) to map which hippocampal networks are activated during rest in PD and to determine if they differ from HC, (2) to explore if reduced AHA within these networks occurs during rest, and (3) to investigate the implications of reductions of AHA on memory and olfactory performance in PD.

## Materials and methods

### Participants

Forty-four patients with idiopathic PD, as defined by the UK Brain Bank criteria, were purposively recruited from the department of Neurology at Linköping University Hospital^19^. All patients experienced ‘on-off phenomena’, meaning rapid motor fluctuations during the dosing cycle^13^. Consequently, all PD participants were awaiting evaluation for DAPT. A movement disorder specialist examined all PD patients to rule out other neurological disorders than idiopathic PD, psychiatric diagnosis, and apparent cognitive, balance, swallowing or speech impairments. Additionally, forty-seven age-matched healthy controls (HC) with self-reported good mental and physical health were recruited through advertisements and snowball sampling.

Exclusion criteria for both groups included: reported active colds, allergies, or COVID-19 infection; previous nasal cavity surgery; loss of smell assessed by a passive smelling test; mandibular or magnetic/electromagnetic implants; smoking; substantial movement artifacts; and incomplete fMRI-session. In the PD group, participant exclusion occurred due to substantial movement artifacts identified during outlier detection (1), as well as incomplete resting-state functional MRI (rs-fMRI) session (4), resulting in a final PD study group of 39 participants (*n* = 39). In the HC group, one participant was excluded due to an active COVID-19 infection, resulting in final HC study group of 46 participants (*n* = 46). These sample sizes were consistent with previous related studies using similar resting-state fMRI methodologies and co-activation pattern (CAP) analysis in clinical populations^20^. All participants were recruited between 2020 and 2023 and provided informed consent prior to the study, which was approved by the Swedish Ethical Review Authority (registration number 2019–02679). All methods and procedures used in this study were performed in accordance with relevant institutional guidelines and regulations. Participants’ demographic data, performance in memory and olfactory assessment, and the number of removed frames from analysis are presented in Table 1.

### Memory and olfactory assessment

Only PD patients completed the memory and olfactory assessment, which included four tests conducted in the same order as presented here. First, the Swedish version of the Rey Auditory Verbal Learning Test (RAVLT) was administered, featuring a wordlist of 15 target words. PD patients were asked to recall the wordlist after each trial (5 trials in total), once after memorizing and recalling a wordlist with 15 distractor words (immediate recall) and once after 30 min (delayed recall). Following the delayed recall, patients completed a recognition task, recognizing the target words from a list of 50 words (15 target and 35 distractor words)^21^.

Second, the Brief Visuospatial Memory Test-Revised (BVMTR) was administered, featuring 6 simple target figures in a 2 × 3 array. PD patients were instructed to recall as many figures as possible at their proper locations after a 10 s learning phase (3 trials in total comprising the immediate recall score), and once after 30 min (delayed recall). Following the delayed recall, patients performed a recognition task, recognizing the target figures from stimulus booklet containing 12 figures (6 target and 6 distractor figures)^21^.

Third, an odor identification task from the test Sniffin’ Sticks was conducted. Sixteen everyday odors were presented with pen-like devices in the following sequence: licorice, pineapple, orange, banana, rose, apple, cinnamon, anise, fish, coffee, leather, clove, peppermint, lemon, garlic and turpentine. After each presentation, PD patients were asked to select one verbal label, the one describing the presented odor most accurately, from four alternative answers. Odors were presented for 3 s, and an odorless pause of 30 s between presentations was taken to avoid habituation^22^.

Fourth, the General Ability Index (GAI), a composite measure of global intellectual ability that is less influenced by fine motor skills, working memory, and processing speed, and the Digit Span (DS), which assesses working memory, were derived from the Wechsler Adult Intelligence Scale-Fourth Edition (WAIS-IV). The DS task included three subtests: DS forward, DS backward, and DS sequencing. In DS forward, PD patients were asked to repeat a sequence of numbers exactly as the examiner read them. In DS backward, the sequence of numbers was repeated in reverse order. In DS sequencing, the sequence of numbers was repeated in ascending numeric order. For all subtests, each correct answer resulted in the presentation of a longer sequence of numbers until the participant failed in two sequences at the same length^21^.

### MRI acquisition

MRI scans were performed using a 3 T Siemens MAGNETOM Prisma scanner (Siemens AG, Erlangen, Germany) equipped with a 64-channel head-neck coil. Prior to rs-fMRI, high-resolution structural T1-weighted 3D volume acquisitions were obtained, repetition time [TR] = 2300 ms; echo time [TE] = 2.36 ms; inversion time [TI] = 900 ms; flip angle = 8°; field of view [FOV] = 250 × 250 mm^2^; slice thickness (gap) = 0.9 (0) mm, and voxel size = 0.9 × 0.9 × 0.9 mm. These structural images were later co-registered with functional T2*-weighted images, which utilized blood oxygenation level-dependent (BOLD) contrast. A multiplex echo planar imaging (EPI) sequence with an initial fat saturation pulse was employed, featuring the following parameters: repetition time [TR] = 878 ms; echo time [TE] = 24 ms; flip angle = 56°; integrated parallel acquisition technique [iPAT] = 3; EPI factor = 68, field of view [FOV] = 204 × 204 mm²; number of slices = 45; slice thickness (gap) = 3 (0) mm, and voxel size = 3 × 3 × 3 mm³. Prior to the rs-fMRI, participants were instructed to remain awake and to focus their gaze on a white cross displayed against a black background through goggles. The rs-fMRI session lasted for 10 min and generated 680 frames as data points. The time interval between the fMRI session and the memory and olfactory assessment did not exceed one month.

### Image preprocessing

For the preprocessing of the rs-fMRI data, we utilized the FMRIB Software Library (FSL) version 6. Brain tissue was extracted from each participant’s structural images. Motion estimates were calculated, and linear transformations were performed for three translation and three rotation head motion parameters. Spatial smoothing was applied to the functional images using a 5 mm full width at half maximum (FWHM) Gaussian kernel. Subsequently, corrected functional images were co-registered to high-resolution structural images applying brain boundary registration methodology. For normalization, structural and functional images were non-linearly warped to the Montreal Neurological Institute (MNI) space. Band-pass filtering, retaining frequencies between 0.01 Hz and 0.1 Hz, was completed using the Data Processing & Analysis for Brain Imaging (DPABI) toolbox version 8.2. Poor registration results, defined as absolute head motion greater than 3 mm or relative head motion greater than 1.5 mm, led to exclusion from the study. Additionally, frames with displacement level greater than 0.5 mm were automatically excluded during the co-activation pattern (CAP) analysis based on a selected movement distortion threshold.

### Co-activation pattern analysis

To examine whether hippocampal asymmetric activity during rest differed between HC and PD groups, the right and left hippocampus were extracted from the Neuromorphometrics atlas included in the Computational Anatomy Toolbox (CAT12) and used as seeds in the CAP analysis^23^. This analytical and data-driven approach was selected for its exploratory nature and capacity to capture dynamic and transient hippocampal configurations over time during rest, aligning with the conceptualization of memory as a multifaceted cognitive function involving diverse neural processes and demands^16^. Frames included in the analysis were selected based on the hippocampal activity threshold (either one or both seeds demonstrated an activity T > 1) and movement distortion level (M < 0.5 mm framewise displacement)^24–26^.

To determine the optimal number of clusters for decomposing the retained data, consensus clustering was applied^27^. A range of K = 1 to 10 was explored. Data from the HC group was repeatedly subsampled (80%, 25 iterations) and clustered to yield a consensus matrix. Consensus values of 0/1 highlighted pairs of frames that were never/always clustered together, indicating stability. The percentage of ambiguously clustered pairs of frames (PAC; for which consensus took intermediate values) was used as a metric for clustering quality, resulting in the optimal number of clusters, hence the optimal number of CAPs/Networks (smallest PAC)^28^.

For each resting-state scan, time points were labeled as reflective of either a Network or baseline hippocampal configuration. This labeling was based on whether one or both seeds demonstrated an activity level above the threshold, along with simultaneous strong and consistent activity in other brain areas. The term ‘expression’ is used throughout the text to denote above-threshold activity for both hippocampal activations and Networks. For each Network, we computed entries (number of times a given Network was entered) and duration (mean time in sec a Network was expressed). Additionally, to examine hippocampus lateralization, the number of frames associated with left hippocampus and right hippocampus were extracted for each group and for each Network.

### Statistics

To determine if the PD group differed from the HC group regarding age, sex and the number of removed frames from analysis, we employed t-tests and chi-squared tests, refer to Table 1 for detailed demographic data and group differences. Subsequently, we conducted a logistic regression analysis with group affiliation as the binary outcome variable (PD group = 1, HC group = 0). Our model included 13 predictors: age, sex, number of removed frames, and entries and duration for each generated Network (5 Networks in total, resulting in 10 predictors). For further details on predictors and their significance for group affiliation, see Table 2. We then extracted the mean number of expressions of the left and the right hippocampus for each Network. These values were compared in a paired t-test with Bonferroni correction to investigate hippocampus lateralization within each group and each Network, see Table 3. Additionally, a lateralization index score for each Network, ranging from − 100 (totally left) to 100 (totally right), was computed based on the Hemispheric Asymmetry Reduction in Older Adults (HAROLD) model, comparing between right vs. left region of interest (ROI)^15^:

$$[(rightROI-leftROI)/(rightROI+leftROI)]\times100.$$

For detailed information on lateralization index scores for each Network and group, refer to Table 3.

To explore the role of asymmetric hippocampal activity (AHA) in memory and odor identification performance, the PD group was divided into two subgroups based on lateralization index scores for each network: PD-AHA^+^, comprising patients with scores deviating ± 1 SD from the PD group mean (i.e., increased AHA), and PD-AHA^−^, comprising those with scores within ± 1 SD of the mean (i.e., reduced AHA).To investigate differences in performance on odor identification and memory tests between the PD-AHA^+^ and PD-AHA^−^ subgroups, we conducted an exploratory, hypotheses-generating multivariate analysis of covariance (MANCOVA). To enhance the robustness of the model and account for potential sources for variability, we included multiple covariates: sex, handedness, age, motor symptoms onset side, levodopa equivalent daily dose, number of years since motor symptoms onset and number of removed frames from analysis. To preserve the natural variability inherent in the behavioral data and to enhance reproducibility, all data points were retained in this analysis. For detailed MANCOVA results, refer to Table 4. All statistical analyses, apart from the extraction of CAP metrics, were performed using IBM SPSS Statistics, version 29.

## Results

### Groups characteristics

The PD group experienced “on-off phenomena” was indicated, given the group’s mean age ($\:\stackrel{-}{x}=$ 60.13 ± 7.29 years), mean levodopa equivalent daily dose ($\:\stackrel{-}{x}=$ 1050.23 ± 350.33 mg), and mean duration since motor symptoms onset ($\:\stackrel{-}{x}$ *=* 8.82 ± 3.63 years). Approximately 15% of patients with PD reported and demonstrated left-handedness during the memory and olfactory assessment. The mean general ability index (GAI), reflecting global cognitive function, was within the manual-defined normative range ($\:\stackrel{-}{x}$ *=* 97.85 ± 15.16). This was similar to the PD group’s performance in all the memory tests, except for the odor identification test^29^. In contrast, their odor identification performance was significantly below average, with raw scores ranging from 2 to 13 correctly identified odors^22^.

**Table 1:** Demographic data and comparison between parkinson’s disease group and healthy controls group.

Variable	PD (*n* = 39, SD)	HC (*n* = 46, SD)	*P* value
Age (years)	60.13 (± 7.29)	60.30 (± 6.13)	0.905
Males/Females	23/16	16/30	0.026*
Left/right handedness	6/33	-	-
Left/right motor symptom onset	19/20	-	-
PD duration (years)	8.82 (± 3.63)	-	-
LEDD (mg/day)	1050.23 (± 350.33)	-	-
GAI (index score)	97.85 (± 15.16)	-	-
Odor identification	7.00 (± 2.65)	-	-
RAVLT immediate	7.66 (± 3.45)	-	-
RAVLT delayed	7.71 (± 3.38)	-	-
RAVLT recognition	13.07 (± 1.72)	-	-
BVMT-R immediate	18.02 (± 7.80)	-	-
BVMT-R delayed	7.46 (± 2.88)	-	-
BVMT-R recognition	5.53 (± 0.71)	-	-
DS forward	8.79 (± 2.14)	-	-
DS backward	7.87 (± 1.67)	-	-
DS sequencing	7.64 (± 2.00)	-	-
Frames removed	22.53 (± 27.73)	5.89 (± 12.92)	0.001*

PD, Parkinson’s Disease; HC, healthy controls; SD, standard deviation; LEDD, levodopa equivalent daily dose; GAI, General Ability Index; RAVLT, Rey Auditory Verbal Learning Test; BVMT-R, Brief Visuospatial Memory Test – Revised; immediate, immediate recall; delayed, delayed recall; DS, Digit Span; *, significant outcome.

The PD group and the HC group did not differ regarding age (*t* (83) = −0.121, *p* = 0.905). However significant differences were observed in sex distribution (χ^2^ (1, *N* = 85) = 4.98, *p* = 0.026) and the number of removed frames from analysis, significantly more frames were excluded for PD compared to the HC group due to frames displacement level (*t* (83) = 3.635, *p* = 0.001). For more details refer to Table 1.

Hippocampal co-activation patterns.

**Fig. 1:**
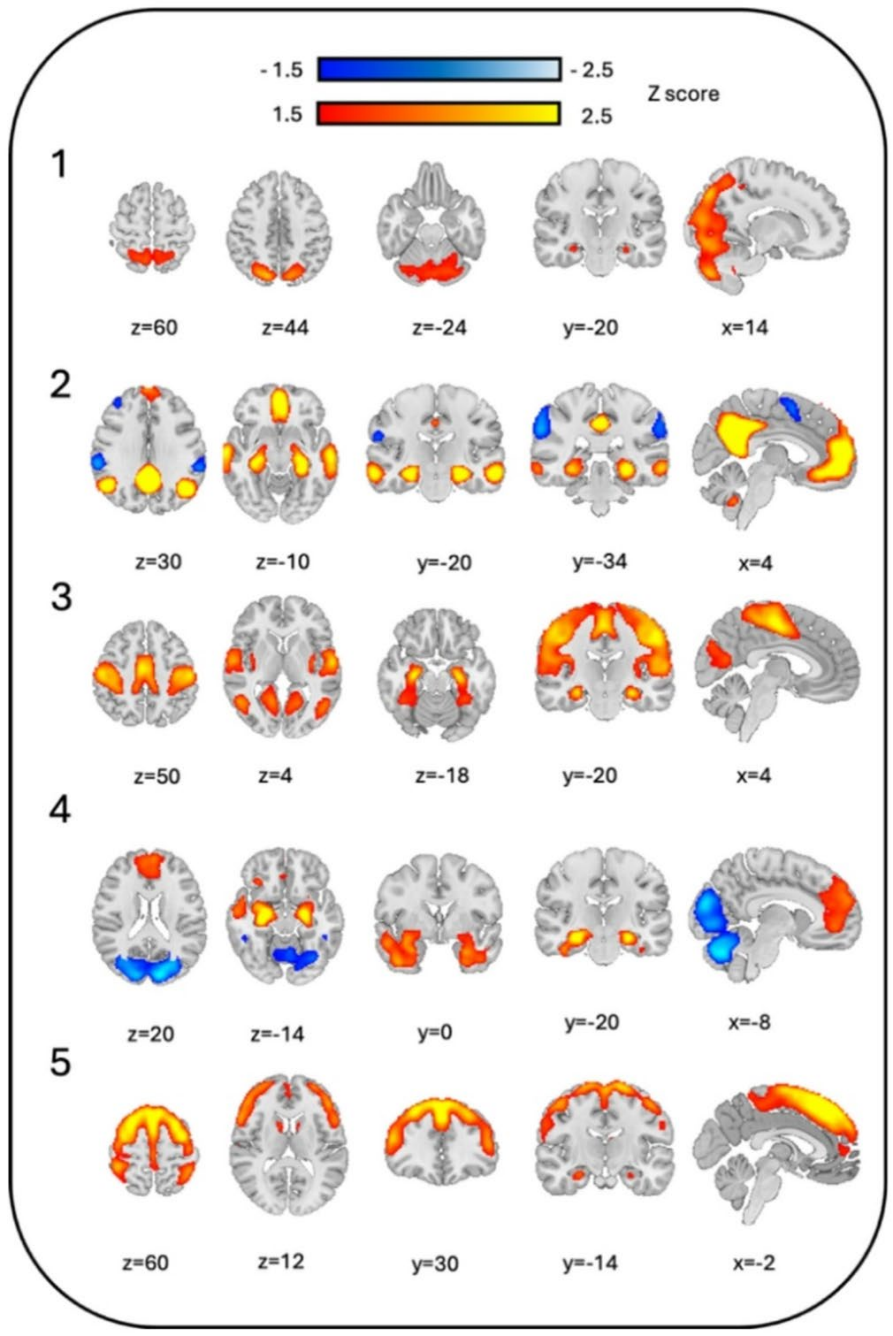
Illustration of the generated hippocampal co-activation patterns generated from healthy controls: (1) Network 1 incorporating the visual cortex, cerebellum, superior parietal lobule, and precuneus. (2) Network 2 incorporating parts of the default mode network with dissociation between precuneus, posterior cingulate gyrus, prefrontal cortex, and middle temporal gyrus (positive), and angular gyrus, supramarginal gyrus, and middle frontal gyrus (negative). (3) Network 3 incorporating precentral gyrus, postcentral gyrus, supplementary motor area (SMA), posterior insula, fusiform gyrus, lingual gyrus, and visual cortex. (4) Network 4 incorporating a dissociation between prefrontal cortex, anterior cingulate gyrus, piriform cortex, amygdala, entorhinal cortex, olfactory cortex, and middle temporal gyrus (positive) and visual cortex, and cerebellum (negative). (5) Network 5 incorporating parts of the central executive network including superior parietal lobule, superior, middle, and inferior frontal gyrus, caudate nucleus, precentral gyrus, and SMA.

Frames were automatically excluded from analysis due to high movement distortion level (M > 0.5 mm) or low hippocampal activity level (T < 1). The percentage of remained frames for analysis after exclusion was approximately 20% for both groups. The optimum number of clusters after consensus clustering was 5. Hence, 5 hippocampal Networks were generated and explored. All the hippocampal Networks are presented in Fig. 1.

Network 1 included the visual cortex, cerebellum, superior parietal lobule, and precuneus. Network 2 incorporated parts of the default mode network (DMN), showing a dissociation between precuneus, posterior cingulate gyrus, prefrontal cortex, and middle temporal gyrus (positive), and angular gyrus, supramarginal gyrus, and middle frontal gyrus (negative). Network 3 included precentral gyrus, postcentral gyrus, supplementary motor area (SMA), posterior insula, fusiform gyrus, lingual gyrus, and visual cortex. Network 4 showed a dissociation between prefrontal cortex, anterior cingulate gyrus, piriform cortex, amygdala, entorhinal cortex, olfactory cortex, and middle temporal gyrus (positive) and visual cortex, and cerebellum (negative). Network 5 incorporated parts of the central executive network (CEN), including superior parietal lobule, superior, middle, and inferior frontal gyrus, caudate nucleus, precentral gyrus, and SMA.

Our logistic regression analysis revealed that most of the Networks in terms of the number of activities (entries) and the mean activity time (duration) were not significantly associated with the presence of PD. However, the duration of Network 1 was significantly related with PD presence, with a beta coefficient of −8.349 (*p* = 0.004), indicating a strong negative relationship, i.e. longer activity duration in Network 1 substantially decreased the likelihood of having PD. Similarly, the number of entries into Network 5 showed a significant negative association with PD presence. The beta coefficient of −0.149 (*p* = 0.009) indicated that more frequent entries into Network 5 decreased the likelihood of having PD by 13.9%. Age, sex and the number of removed frames were not significantly associated with PD presence. The overall model was statistically significant, demonstrating a substantial improvement over a model without predictors (χ^2^(13) = 43.426, *p* < 0.001) and explaining 53% of the variance in the data (Pseudo R^2^ = 0.535). Furthermore, most predictors in the regression analysis exhibited variance inflation factor (VIF) values between 1.2 and 2.1, indicating low multicollinearity among predictors. Only one predictor, the number of removed frames, had a VIF of 5.3, reflecting a moderate level of multicollinearity that can be considered as acceptable, particularly given that a significant group difference observed between PD and HC regarding this variable. For more details, refer to Table 2.

**Table 2:** Logistic regression analysis predicting the presence of parkinson’s disease.

Predictor	B	SE	Z ratio	*P* value	Odds ratio	95% CI for Exp(B)
Age	0.035	0.046	0.569	0.451	1.035	0.946–1.133
Sex	0.883	0.693	1.625	0.202	2.419	0.622–9.408
Removed frames	−0.015	0.033	0.213	0.645	0.985	0.924–1.050
Network 1						
Entries	−0.076	0.054	1.934	0.164	0.927	0.833–1.031
Duration	−8.349	2.916	8.196	0.004*	0.000	0.000–0.072
Network 2						
Entries	−0.038	0.053	0.508	0.476	0.963	0.869–1.068
Duration	1.764	1.294	1.859	0.173	5.835	0 0.462 − 73 0.652
Network 3						
Entries	−0.078	0.069	1.278	0.258	0.925	0.807–1.059
Duration	−2.127	1.483	2.059	0.151	0.119	0 0.007 − 2 0.178
Network 4						
Entries	−0.055	0.038	2.069	0.150	0.947	0.879–1.020
Duration	−1.359	1.959	0.481	0.488	0.257	0 0.006–11.954
Network 5						
Entries	−0.149	0.058	6.754	0.009*	0.861	0.769–0.964
Duration	−2.114	2.247	0.885	0.347	0.121	0.001–9.885
Constant	18.032	8.070	4.993	0.025	-	-

B, beta coefficient; SE, standard error; CI, confidence interval; Exp, exponential function; *, significant outcome.

### Hippocampal lateralization

Based on initial inspection of Table 3, the PD group exhibited higher lateralization index scores in Network 3, while lower scores were observed in Network 4 and 5. In contrast, the HC group showed the opposite pattern with lower scores in Network 3 and higher scores in Network 4 and 5, accompanied by a predominance of right hippocampal activity. Paired t-test comparing right and left hippocampal activity within each network and group showed no significant differences in the PD group. However, the HC group exhibited significant differences within two Networks: Network 2 with greater left hippocampal activity (*p* = 0.010) and Network 4 with greater right hippocampal activity (*p* = 0.040). For more information, refer to Table 3.

**Table 3:** Lateralization index and comparison between the number of left vs. right hippocampus expressions within each group related to each network.

Group	Network	Lateralization index (SD)	Right hippocampus (SD)	Left hippocampus (SD)	Right vs. Left hippocampus *P* value corr.
PD	Network 1	−4.98 (± 31.36)	7.61 (± 3.78)	8.64 (± 5.06)	1.000
	Network 2	−12.36 (± 28.33)	8.71 (± 6.16)	10.46 (± 6.99)	0.170
	Network 3	11.33 (± 35.63)	7.84 (± 4.10)	6.53 (± 4.21)	0.560
	Network 4	−0.60 (± 36.61)	7.30 (± 4.87)	6.71 (± 4.14)	1.000
	Network 5	0.97 (± 43.35)	4.46 (± 3.04)	4.25 (± 2.57)	1.000
HC	Network 1	−9.03 (± 31.92)	9.00 (± 4.74)	10.76 (± 5.00)	0.370
	Network 2	−16.15 (± 30.23)	8.86 (± 6.26)	11.50 (± 6.61)	0.010*
	Network 3	0.71 (± 27.41)	8.26 (± 4.13)	8.47 (± 4.59)	1.000
	Network 4	15.99 (± 32.58)	9.78 (± 5.62)	7.23 (± 5.10)	0.040*
	Network 5	8.76 (± 30.66)	7.41 (± 3.78)	6.23 (± 3.04)	0.490

PD, Parkinson’s disease; HC, healthy controls; SD, standard deviation; corr., Bonferroni-corrected (5 networks x 2 groups = 10); *, significant outcome.

A MANCOVA analysis was conducted to examine the effect of AHA status (PD-AHA^+^ vs. PD-AHA^−^) within each Network on multiple memory and odor identification tests, controlling for age, sex, handedness, motor symptoms onset side, levodopa equivalent daily dose, PD duration, and the number of removed frames from analysis. Multivariate results indicated that AHA in none of the examined networks had a significant overall effect on the combined memory and olfactory performance: Network 1 (Wilks’ Lambda = 0.662, F (10,21) = 1.073, *p* = 0.423), Network 2 (Wilks’ Lambda = 0.731, F (10,21) = 0.774, *p* = 0.652), Network 3 (Wilks’ Lambda = 0.502, F (10,21) = 2.084, *p* = 0.075), Network 4 (Wilks’ Lambda = 0.680, F (10,21) = 0.989, *p* = 0.482), and Network 5 (Wilks’ Lambda = 0.596, F (10,21) = 1.426, *p* = 0.236).

However, univariate analyses demonstrated significant effects of AHA within three Networks. AHA within Network 1 (24 PD-AHA^−^, 15 PD-AHA^+^) significantly affected the performance in the RAVLT recognition subtest (F (1,30) = 6.505, *p* = 0.016). Additionally, AHA showed a tendency to affect performance in two other auditory-verbal memory subtests: RAVLT immediate recall (F (1,30) = 3.842, *p* = 0.059), and delayed recall (F (1,30) = 3.958, *p* = 0.056). AHA within Network 3 (26 PD-AHA^−^, 13 PD-AHA^+^) significantly affected the performance in the DS sequencing subtest (F (1,30) = 9.444, *p* = 0.004). AHA within Network 5 (29 PD-AHA^−^, 10 PD-AHA^+^) significantly affected the performance in the odor identification test (F (1,30) = 6.782, *p* = 0.014), and the DS forward subtest, (F (1,30) = 5.399, *p* = 0.027). These results were not corrected for multiple comparisons and did not reach the Bonferroni-adjusted significance threshold of *p* < 0.001, which accounts for 50 tests (5 networks x 10 behavioral tests).

In summary, the PD-AHA^+^ subgroups outperformed the PD-AHA^−^ subgroups, indicating a relationship between increased AHA and better performance across multiple domains, including auditory-verbal recognition memory, auditory-verbal short-term memory, auditory-verbal working memory, auditory-verbal long-term memory, attention and odor identification. For more details, refer to Table 4.

**Table 4:** Comparison between PD patients with and without reduced asymmetric hippocampal activity related to each memory test, controlling for sex, handedness, age, motor symptoms onset side, Levodopa equivalent daily dose, number of years since motor symptoms onset and number of removed frames.

Network Subgroup (*n*)	Test	PD-AHA^−^ (SD)	PD-AHA^+^ (SD)	Mean square	F (1, 30)	*P* value uncorr.
Network 1						
PD-AHA^−^, (*n* = 24) PD-AHA^+^, (*n* = 15)	Odor identification	6.95 (± 2.82)	7.06 (± 2.46)	0.411	0.055	0.816
	RAVLT immediate	6.70 (± 3.22)	9.20 (± 3.36)	41.334	3.842	0.059
	RAVLT delayed	6.91 (± 3.10)	9.00 (± 3.52)	37.986	3.958	0.056
	RAVLT recognition	12.54 (± 1.88)	13.93 (± 0.96)	14.955	6.505	0.016*
	BVMT-R immediate	17.33 (± 8.45)	19.13 (± 6.77)	3.418	0.070	0.794
	BVMT-R delayed	7.00 (± 3.00)	8.20 (± 2.59)	7.829	1.285	0.266
	BVMT-R recognition	5.37 (± 0.82)	5.80 (± 0.41)	1.725	2.992	0.094
	DS forward	8.70 (± 2.19)	8.93 (± 2.12)	0.390	0.008	0.930
	DS backward	8.00 (± 1.53)	7.66 (± 1.91)	2.006	0.630	0.434
	DS sequencing	7.50 (± 2.04)	7.86 (± 1.99)	1.067	0.230	0.635
Network 2						
PD-AHA^−^, (*n* = 30) PD-AHA^+^, (*n* = 9)	Odor identification	7.23 (± 2.78)	6.22 (± 2.10)	3.429	0.468	0.499
	RAVLT immediate	7.66 (± 3.47)	7.66 (± 3.60)	0.021	0.002	0.967
	RAVLT delayed	7.56 (± 3.33)	8.22 (± 3.70)	1.411	0.130	0.721
	RAVLT recognition	12.96 (± 1.86)	13.44 (± 1.13)	6.541	2.536	0.122
	BVMT-R immediate	17.73 (± 8.24)	19.00 (± 6.46)	1.789	0.036	0.850
	BVMT-R delayed	7.33 (± 2.95)	7.88 (± 2.75)	1.416	0.225	0.639
	BVMT-R recognition	5.50 (± 0.77)	5.66 (± 0.50)	0.319	0.512	0.480
	DS forward	8.73 (± 2.31)	9.00 (± 1.50)	3.425	0.713	0.405
	DS backward	7.73 (± 1.61)	8.33 (± 1.87)	1.379	0.430	0.517
	DS sequencing	7.53 (± 2.17)	8.00 (± 1.32)	0.591	0.127	0.724
Network 3						
PD-AHA^−^, (*n* = 26) PD-AHA^+^, (*n* = 13)	Odor identification	6.92 (± 2.77)	7.15 (± 2.51)	6.048	0.835	0.368
	RAVLT immediate	7.50 (± 3.59)	8.00 (± 3.29)	16.441	1.419	0.243
	RAVLT delayed	7.53 (± 3.28)	8.07 (± 3.68)	9.408	0.892	0.353
	RAVLT recognition	13.19 (± 1.78)	12.84 (± 1.62)	0.000	0.000	0.993
	BVMT-R immediate	17.38 (± 6.89)	19.30 (± 9.55)	99.910	2.174	0.151
	BVMT-R delayed	7.23 (± 2.98)	7.92 (± 2.72)	18.881	3.299	0.079
	BVMT-R recognition	5.46 (± 0.81)	5.69 (± 0.48)	0.722	1.182	0.286
	DS forward	8.61 (± 2.13)	9.15 (± 2.19)	1.256	0.258	0.615
	DS backward	7.65 (± 1.87)	8.30 (± 1.10)	4.734	1.530	0.226
	DS sequencing	7.00 (± 1.89)	8.92 (± 1.60)	33.594	9.444	0.004*
Network 4						
PD-AHA^−^, (*n* = 25) PD-AHA^+^, (*n* = 14)	Odor identification	7.00 (± 2.84)	7.00 (± 2.38)	0.016	0.002	0.963
	RAVLT immediate	7.60 (± 3.67)	7.78 (± 3.16)	0.000	0.000	0.999
	RAVLT delayed	7.60 (± 3.31)	7.92 (± 3.62)	1.415	0.131	0.720
	RAVLT recognition	12.96 (± 1.85)	13.28 (± 1.48)	1.520	0.553	0.463
	BVMT-R immediate	18.40 (± 7.57)	17.35 (± 8.44)	10.878	0.222	0.641
	BVMT-R delayed	7.56 (± 2.90)	7.28 (± 2.94)	0.005	0.001	0.978
	BVMT-R recognition	5.56 (± 0.76)	5.50 (± 0.65)	0.153	0.243	0.626
	DS forward	8.32 (± 1.95)	9.64 (± 2.27)	17.129	3.942	0.056
	DS backward	7.76 (± 1.61)	8.07 (± 1.81)	0.205	0.063	0.803
	DS sequencing	7.72 (± 2.26)	7.50 (± 1.50)	0.192	0.041	0.841
Network 5						
PD-AHA^−^, (*n* = 29) PD-AHA^+^, (*n* = 10)	Odor identification	6.55 (± 2.52)	8.30 (± 2.71)	41.163	6.782	0.014*
	RAVLT immediate	7.37 (± 3.63)	8.50 (± 2.87)	8.171	0.689	0.413
	RAVLT delayed	7.44 (± 3.38)	8.50 (± 3.43)	12.868	1.233	0.276
	RAVLT recognition	13.17 (± 1.71)	12.80 (± 1.81)	0.034	0.012	0.913
	BVMT-R immediate	17.10 (± 7.01)	20.70 (± 9.66)	58.294	1.231	0.276
	BVMT-R delayed	7.03 (± 2.65)	8.70 (± 3.30)	9.958	1.654	0.208
	BVMT-R recognition	5.48 (± 0.78)	5.70 (± 0.48)	0.540	0.876	0.357
	DS forward	8.62 (± 2.11)	9.30 (± 2.26)	22.494	5.399	0.027*
	DS backward	7.65 (± 1.79)	8.50 (± 1.08)	3.140	0.998	0.326
	DS sequencing	7.37 (± 2.09)	8.40 (± 1.57)	8.845	2.018	0.166

PD, Parkinson’s disease; AHA^+^, increased asymmetric hippocampal activity; AHA^−^, reduced asymmetric hippocampal activity; SD, standard deviation; RAVLT, Rey Auditory Verbal Learning Test; BVMTR, Brief Visuospatial Memory Test – Revised; immediate, immediate recall; delayed, delayed recall; DS, Digit Span; F(1, 30), 1 effect degrees of freedom (df) and 30 error df; uncorr., uncorrected p-values; *, significant outcome.

## Discussion and conclusion

Previous studies on Parkinson’s Disease (PD) using resting-state fMRI (rs-fMRI) have primarily demonstrated a link between cognitive symptoms and reduced connectivity in the default mode network (DMN) and the central executive network (CEN)^30^. Additionally, alternations in the frontostriatal connectivity involving the cholinergic system have been associated with other non-motor symptoms such as REM sleep behavior disorder, autonomic dysfunction, visual hallucination, and olfactory decline. These have been shown to be predictive of the future risk of developing dementia in PD^31^. Furthermore, reduced hippocampal volume and connectivity, especially with posterior supratentorial areas and the cerebellum, have been related to decline in olfactory performance^12^.

Building on these findings, our study explored hippocampal co-activation patterns (CAPs) and lateralization, revealing several novel insights. We focused on differences between the Parkinson’s Disease (PD) group and healthy controls (HC), as well as between PD subgroups with increased (PD-AHA^+^) or reduced (PD-AHA^−^) asymmetric hippocampal activity. The PD subgroup differences based on AHA should be considered exploratory, as they did not remain significant after correction for multiple comparisons. Notably, the likelihood of having PD decreased significantly when hippocampal Network 1 exhibited longer average activity duration and when hippocampal Network 5 was activated more frequently. Network 1 included the visual cortex, cerebellum, superior parietal lobule, and precuneus, while Network 5 consisted of components of the CEN such as the superior parietal lobule, superior, middle, and inferior frontal gyri, caudate nucleus, precentral gyrus, and supplementary motor area. Moreover, the PD group showed a general tendency towards reduced hippocampal expression and lateralization. The occurrence of reduced AHA in PD during rest indicates that this activity pattern is not exclusively task related. While PD patients did not demonstrate AHA in any hippocampal network, the HC group showed AHA in two networks. Network 2 included the middle temporal gyrus and parts of the DMN such as the precuneus, posterior cingulate gyrus, and prefrontal cortex, and Network 4 consisted of the prefrontal cortex, anterior cingulate gyrus, piriform cortex, amygdala, entorhinal cortex, olfactory cortex, and middle temporal gyrus.

Compared to the PD-AHA^+^ subgroups, the PD-AHA^−^ subgroups showed a general tendency towards lower performances in the memory and olfactory assessments. Specifically, significant or nearly significant deficits were demonstrated in relation to the same hippocampal networks that distinguished PD patients from HC, namely Network 1 and Network 5. Network 1, primarily composed of the occipital network (ON) and thus predominantly related to visual processing, was associated with lower performances in tasks measuring auditory-verbal short-term, long-term, and recognition memory among PD-AHA^−^ patients^32^. Interestingly, no significant deficits were observed in visuospatial memory tasks. This can be attributed to the involvement of other regions in Network 1 associated with language processing and postural balance, such as the cerebellum^33^. Moreover, Network 1 was more lateralized towards the left hippocampus, which in right-handed individuals (the majority of the PD group), is closely associated with auditory-verbal memory functions^34^. Furthermore both the ON and precuneus have been related to visual imagery which can serve as a strategy to recall auditory-verbal information^35^. For Network 5, which primarily includes the lateral frontoparietal network (L-FPN) and thus predominantly associated with working memory, executive function, goal-directed cognition, decision-making, and mental shifting, PD-AHA^−^ patients exhibited low performances in tasks measuring auditory-verbal short-term and working memory as well as odor identification^32^. Since Network 5 did not include core olfactory related brain regions apart from the hippocampus, the relation between this network and odor identification performance may be mainly attributed to the test’s decision-making procedure. However, previous studies have shown a distinct association between grey matter volumetric measures and seed-based fMRI alternations in the hippocampus, both of which have been linked to olfactory deficit in PD^12^. Moreover, significant low auditory-verbal working memory performance was demonstrated in relation to Network 3. This hippocampal network includes among other brain regions the precentral gyrus, which is related to working memory and executive functions^36^.

Our study had some limitations, which could impact our findings, and therefore should be addressed. Significant differences were observed between the PD and HC groups regarding sex distribution and the number of removed frames from analysis. However, these were included as predictors in the regression analysis and did not show a significant effect on the outcome variable, the presence of PD. Conversely, the levodopa equivalent daily dose (LEDD) could not be included in this analysis since it is specific to the PD group. Nevertheless, dopaminergic medication is often associated with increased hippocampal activity while the PD group, compared to HC, exhibited the opposite pattern^37^.

The absence of certain data for the HC group, such as cognitive status evaluated by standardized neuropsychological tests and handedness, suggests that our findings should interpreted with caution. However, approximately 15% of the PD patients were left-handed, which is in accordance with prevalence estimates in the general normal population, and by extension, is likely representative of the HC group in our study^38^. Furthermore, data on the PD group’s motor severity level extracted from formal standardized scales was missing. However, All PD patients were assessed to be in the mid- to advanced stages of the disease, experiencing motor fluctuations known as “on-off phenomena”. This classification was supported by both their LEDD levels and duration of symptoms since disease onset. Previous studies have shown that LEDD levels below 600 mg/day are typically associated with early-stage PD, while levels in the range of 2000 mg/day are indicative of late-stage disease^39^. Moreover, disease durations longer than 2.4 years have been linked to mid- and advanced stages of PD^40^. In our PD group, all participants had LEDD levels between 699.9 and 1400.56 mg/day, and none had a disease duration shorter than 5.1 years. These findings are consistent with the assessments made by movement disorder specialists, who referred all PD participants for evaluation for DAPT. Furthermore, both LEDD levels and disease durations were included in the MANCOVA as covariates, to reduce potential confounding effects. Nevertheless, both the uncorrected results for multiple comparisons and the use of standard deviation thresholds to classify PD subgroups in our exploratory analysis highlight the need for cautious interpretation and warrant future investigation. Interestingly, a general trend, though not statistically captured, supported the notion that reduced AHA may be related to poorer performance in auditory-verbal memory tasks and odor identification.

Regarding the comparisons between the PD-AHA^+^ and PD-AHA^−^ subgroups, the small sample sizes limit the reliability and generalizability of our preliminary results. Furthermore, some relevant data, such as disease stage, executive function measures and other biomarkers, were not available. Nevertheless, both the neurological assessments, the performance on working memory tasks, and the cognitive global function score, which fell within the manual-defined normal range, suggest that the executive and cognitive functioning was generally preserved in this PD cohort.

In summary, two hippocampal networks, one predominantly involving the visual cortex and the other including lateral frontoparietal brain regions, demonstrated significantly shorter average activation time and lower activation frequency in PD compared to HC. Furthermore, significant lateralization was observed in two hippocampal networks in the HC group, whereas no significant differences between right and left hippocampal activations were found in PD. Notably, the small PD subgroups with reduced asymmetric hippocampal activity (PD-AHA^−^) compared to those with increased AHA (PD-AHA^+^) during rest exhibited a general trend toward lower performance on auditory-verbal memory and odor identification tests. Future studies with larger samples are needed to confirm the robustness and generalizability of this trend. To our knowledge, this is the first study to link changes in resting-state asymmetric hippocampal activity (AHA) with memory and olfactory performance in PD. Our findings align with the HAROLD model, which, although primarily focusing on prefrontal cortex, posits that reduced hemispheric asymmetry during task performance may reflect compensatory recruitment or neural dedifferentiation associated with aging^15^. However, our study extends this framework by demonstrating that asymmetry reductions also occur during rest and within specific brain networks such as hippocampal. Notably, structural and functional asymmetries are considered a normal feature of healthy brain organization, whereas reduction in asymmetry appears not only age-related but also associated with neurodegenerative processes, as observed in our PD cohort^41^.

In this context, reductions of AHA may serve as a marker for characterizing cognitively vulnerable PD patients who may be at risk for developing cognitive impairment following device-aided Parkinson therapy (DAPT). However, future confirmatory and longitudinal studies with larger PD and HC cohorts assessed for cognitive status using standardized neuropsychological tests, are required to further investigate our preliminary findings, particularly the relation of changes in AHA and other cognitive functions in PD, especially global cognition, as well as the predicative value of baseline AHA for memory and overall cognitive status following DAPT. Another important avenue for future investigation is the association between the side of motor symptom onset and AHA, which could help clarify whether changes in AHA reflect disease progression or compensatory mechanism.

## Data Availability

The data supporting our findings is available from the corresponding author, upon reasonable request.
